# Spatiotemporal Analysis of Intranuclear Inclusion Body and Virus Formation in Human Adenovirus Type 5-Infected Cells

**DOI:** 10.3390/ijms27209181

**Published:** 2026-10-16

**Authors:** Hong Wu, Yoshihiko Fujioka, Shoichi Sakaguchi, Chinami Odake, Youichi Suzuki, Takashi Nakano

**Affiliations:** 1 Department of Microbiology and Infection Control, Faculty of Medicine, Osaka Medical and Pharmaceutical University, 2-7 Daigaku-machi, Takatsuki 569-8686, Osaka, Japan; yoshihiko.fujioka@ompu.ac.jp (Y.F.); shoichi.sakaguchi@ompu.ac.jp (S.S.); chinami.odake@ompu.ac.jp (C.O.); tnakano@ompu.ac.jp (T.N.); 2 Laboratory of Biosafety Research, Faculty of Medicine, Osaka Medical and Pharmaceutical University, 2-7 Daigaku-machi, Takatsuki 569-8686, Osaka, Japan

**Keywords:** human adenovirus type 5, intranuclear inclusion body, three-dimensional reconstruction, transmission electron microscopy, electron tomography

## Abstract

Human adenovirus type 5 (HAdV-5) forms a characteristic intranuclear inclusion body in infected cells. However, the relationship between the intranuclear inclusion body and virus formation in HAdV-5-infected cells remains unclear. This study conducted a detailed investigation of the relationship between the structure of the inclusion body and the localization of the virus in HAdV-5-infected cells using electron microscopy and three-dimensional (3D) tomography analytical techniques. Immunostaining for viral proteins was also performed to determine their colocalization with the intranuclear inclusion body. 3D analysis revealed the intranuclear inclusion body as the major site of viral particle accumulation in the cells. Viral particles were identified within the intranuclear inclusion body and arranged in a crystalline structure parallel to the fibrous structures of this body. Moreover, mature viral particles were predominantly distributed around the intranuclear inclusion body. Certain viral proteins were also detected in the intranuclear inclusion body by immunoelectron microscopy. We propose that intranuclear inclusion bodies serve as sites of viral replication in HAdV-5-infected cells. Through the 3D analysis, we clarified the relationship between the distribution and localization of intranuclear inclusion bodies and viral particles in HAdV-5-infected cells. These findings can improve our understanding of adenoviral infection mechanisms and may contribute toward developing novel antiviral strategies.

## 1. Introduction

Human adenoviruses (HAdVs) are pathogens that infect the respiratory, ocular, intestinal, and urinary tracts, with certain types causing severe illness [1,2,3]; in particular, immunocompromised individuals are at high risk for disseminated infection and potentially fatal outcomes [4,5]. Nevertheless, no specific cure is currently available [6].

Adenoviruses are nonenveloped viruses characterized by an icosahedral virion of ~95 nm in size, comprising primarily capsid hexons, and whose vertices are formed by penton base and fiber proteins. The viral genome is a linear, double-stranded DNA that spans 25–46 kb and encodes the early and late genes essential for replication and virion assembly. After entering the host cell via receptor-mediated endocytosis, the virion disassembles, and the nucleocapsid moves to the nucleus, where early gene products are synthesized to modulate the host environment and initiate viral DNA replication. Subsequent expression of late genes results in the production of structural proteins, including capsid, penton, and fiber, and the assembly of new virions in the nucleus. Eventually, progeny viruses are released from the infected cells via lysis, thus completing the replication cycle [7,8].

Although this virus replicates within the cell nucleus, the detailed processes involved have not been clarified [9,10,11]. Intranuclear inclusion bodies are formed in the nuclei of cultured cells infected with HAdV during the late stages of the viral life cycle [12,13,14,15]. Intranuclear inclusion bodies can be readily observed using conventional transmission electron microscopy (TEM) with standard ultrathin sectioning techniques using uranyl acetate and lead citrate staining. They have been reported as intranuclear crystalline structures [14] and can also be readily identified by Giemsa staining under light microscopy [15]. Electron microscopy studies have reported that the fiber knobs and portions of the shaft domain of human adenovirus type 5 (HAdV-5) are essential for the formation of protein crystals in infected cells, which constitute intranuclear inclusion bodies [16]. Moreover, immunofluorescence studies using a monoclonal antibody against the HAdV-5 fiber knob demonstrated strong reactivity with nuclear protein crystals. Furthermore, the absence of intranuclear inclusion bodies in infected cells correlates with reduced infectivity and viral replication [16]. These findings suggest the crucial role of protein crystals in virion assembly and the viral replication cycle.

Nevertheless, the microstructures of these intranuclear inclusion bodies and their relationship with mature virion formation remain poorly understood. Recent advances in three-dimensional (3D) tomography methods have enabled detailed analyses of viruses and their developmental stages [1,2,3,4,5,6,7,8,9,10,11,12,13,14,15,16,17]. In this study, we used 3D reconstructions generated from conventional TEM and electron tomography (ET) to explore the ultrastructural morphology of intranuclear inclusion bodies in HAdV-5-infected Vero cells. Through this approach, we could examine the relationship between the intranuclear inclusion body and virus formation in unprecedented detail in this study. This innovative observation technique helped us gain new insights into the ultrastructure of adenoviral assemblies.

## 2. Results

### 2.1. Morphology of Intranuclear Inclusion Body Evaluated by Optical Microscopy and TEM

**Figure 1:**
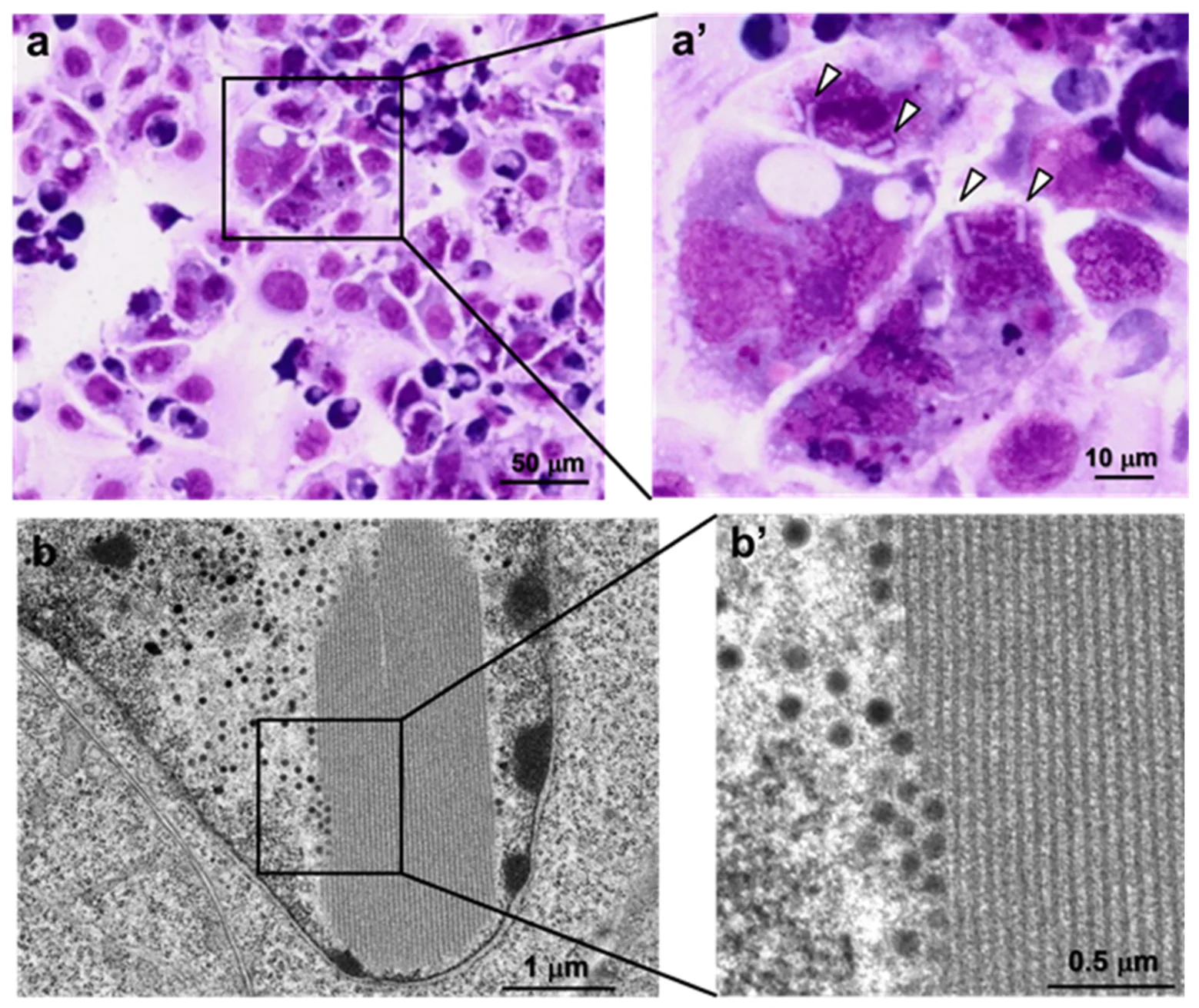
Morphology of intranuclear inclusion bodies in HAdV-5-infected Vero cells observed by light microscopy and TEM. (**a**) Homogeneously stained intranuclear crystal structures were observed by light microscopy after Giemsa staining. (**a’**) A higher magnification micrograph of the region outlined by the black square in (**a**) and the white short arrows) indicate intranuclear crystal structures. Bars = 50 μm in (**a**) and 10 μm in (**a’**). (**b**) Morphology of an intranuclear inclusion body in HAdV-5-infected Vero cells examined by TEM. (**b’**) A higher magnification micrograph of the region outlined by the black square in (**b**). Bars = 1 μm in (**b**) and 0.5 μm in (**b’**).

In our study, we used Vero cells for HAdV-5 infection because they exhibited the highest level of virus replication among the cell lines tested. We first examined the morphology of intranuclear crystal structures in HAdV-5-infected cells by optical microscopy. Homogeneously stained intranuclear crystal structures were detected as shown in Figure 1a, and Figure 1a’ depicts a higher magnification view of the region outlined by the black square in Figure 1a. The arrowheads (white short arrows) indicate intranuclear crystal structures, known as rod-like inclusion bodies. To clarify the details of the intranuclear crystals, we examined the samples by TEM and observed heterochromatic regions within the nucleus of the Vero cell that are crystalline inclusions in the cross-section of HAdV-5-infected Vero cells (Figure 1b); Figure 1b’ depicts a higher magnification view of the region outlined by the black square in Figure 1b. Intranuclear inclusion bodies were observed as aggregates of rectilinear tubular protein filaments in a parallel disposition, and viral particles were closely associated with the intranuclear inclusion body of viral proteins.

### 2.2. Analysis of Intranuclear Inclusion Body and Viral Particles Using 3D Reconstruction of ET Images

**Figure 2:**
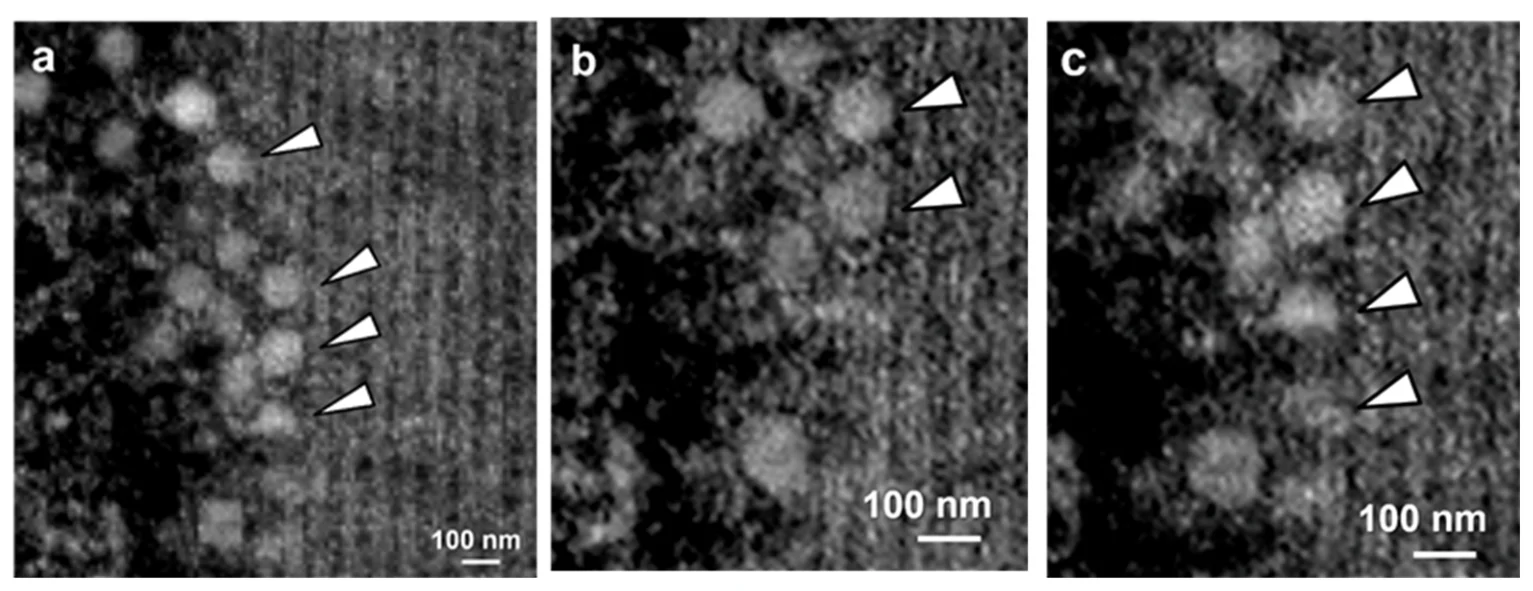
3D ET images of the intranuclear inclusion body in HAdV-5-infected Vero cells. (**a**) 3D ET images of the intranuclear inclusion body in Y and X rotations obtained by ET; the arrowheads indicate the virus-like particles. (**b**,**c**) 3D ET images of the intranuclear inclusion body in the Z-slice position obtained by ET; the arrowheads indicate the virus-like particles. Bars = 100 nm in all images.

To confirm the detailed morphology of the intranuclear inclusion body and its relationship with the viral particles, we performed ET to obtain 3D images from the images depicted in Figure 1b. The 3D images in the Y- and X-directions of Figure 1b demonstrate the intranuclear inclusion body and viral particles. The original video file of the 3D (Y- and X-directions) images obtained by ET is available as Supplementary Video S1. Virus-like particles appeared to protrude from the edge of the intranuclear inclusion body. Figure 2a shows the images of the filaments of the intranuclear inclusion body connecting the virus-like particles (arrowheads) obtained from the best shot to identify certain structures.

The original video file of the 3D (Z-slice position) images obtained by ET is available as Supplementary Video S2. From this slice position, the virus-like particles were also in close contact with the intranuclear inclusion body. Figure 2b,c depicts a representative 3D image of virions protruding from the edge of the filaments in the intranuclear inclusion body obtained from the best shot to identify certain structures. The arrowheads indicate viral particles that appear to emerge from the intranuclear inclusion body. Two virus-like particles (arrowheads in Figure 2b) were detected in the interrupted region of the filaments in the intranuclear inclusion body; however, virus-like particles (arrowheads in Figure 2c) located at a deeper level were also identified. These findings illustrate the presence of virus-like particles, appearing to be from the formation process, in the 3D images within intranuclear inclusion body filaments. The number of viruses varies depending on the depth of the fault at the same location, as depicted in Figure 2b,c in the 3D images obtained by ET. This phenomenon indicates that the virus-like particles are embedded within the filaments of the intranuclear inclusion body structure.

**Figure 3:**
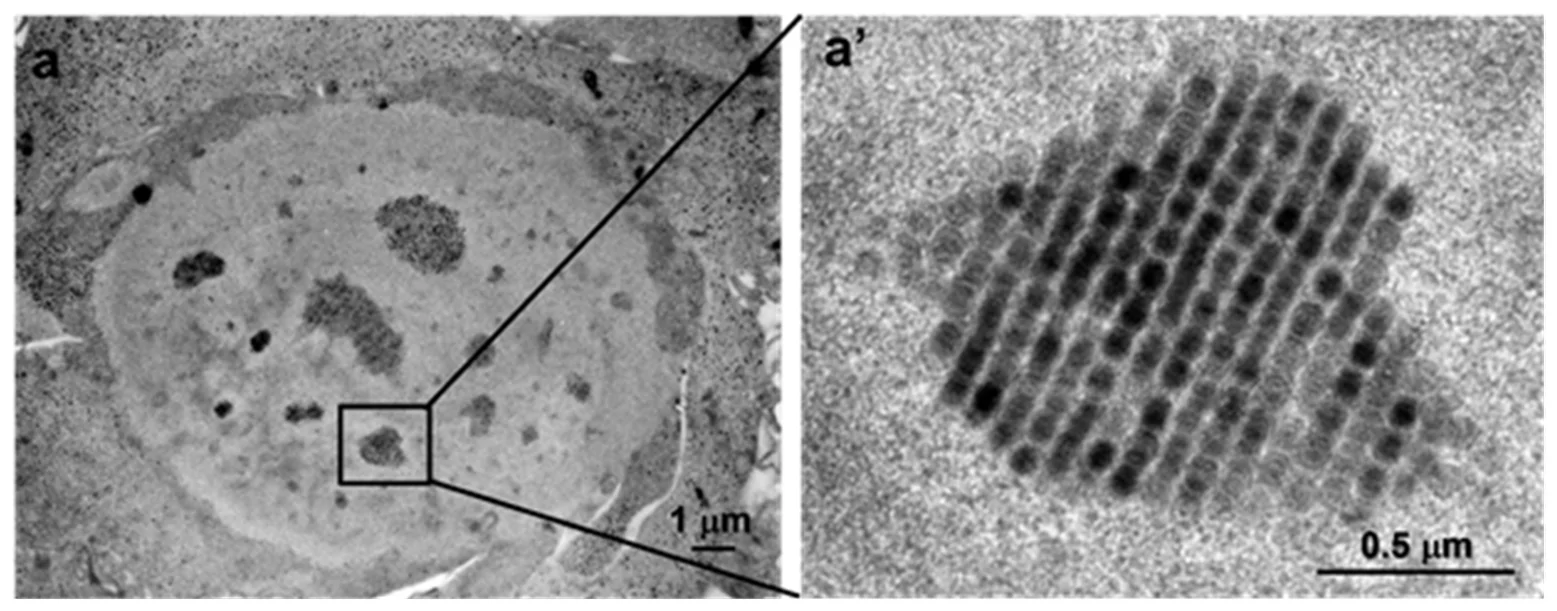

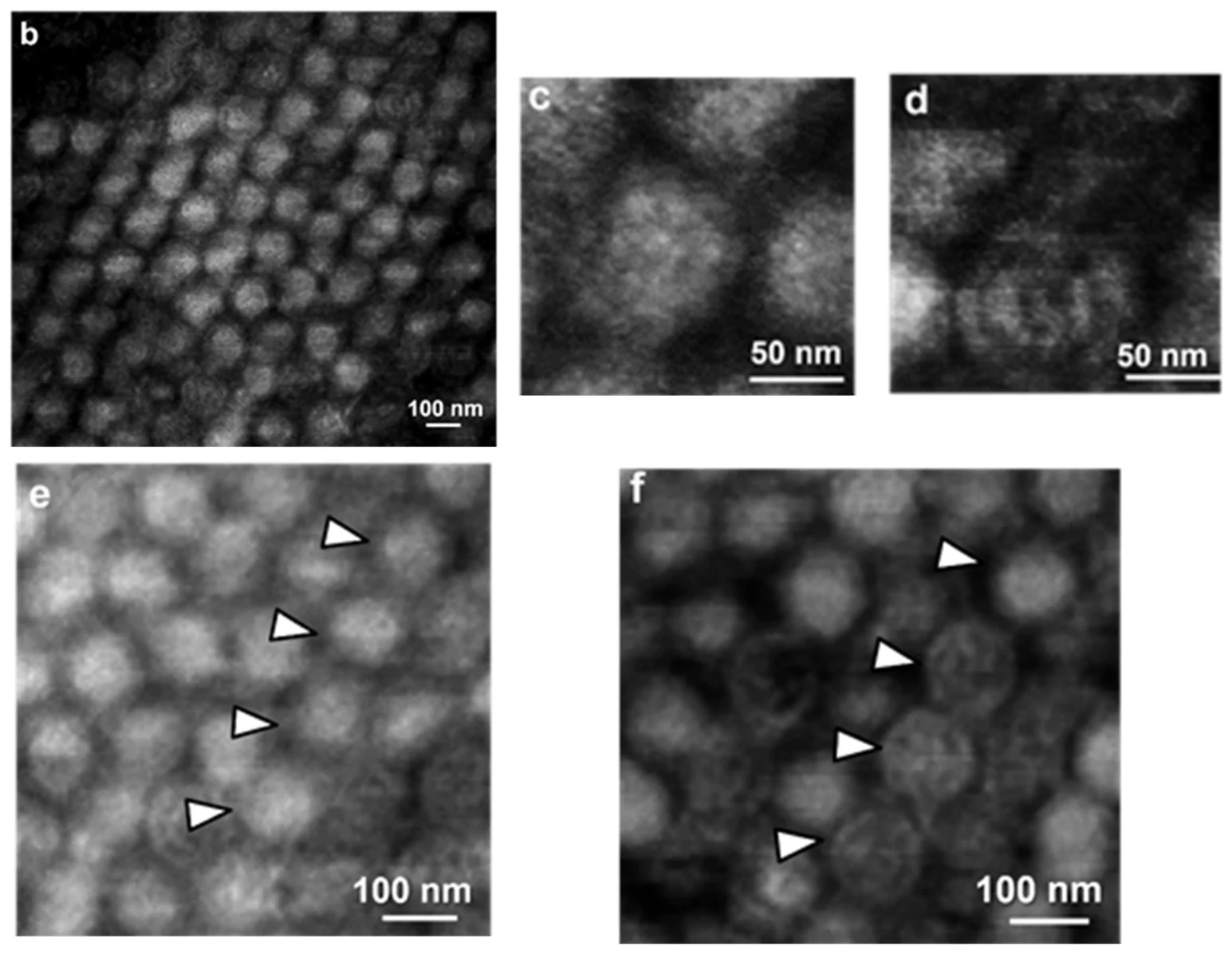
The morphology of the clusters of virus-like particles appears to coincide with traces of intranuclear inclusion body filaments, as in HAdV-5-infected Vero cells, observed by TEM and ET. (**a**) Morphology of the clusters of virus-like particles in HAdV-5-infected Vero cells observed by TEM. (**a’**) A higher magnification micrograph of the region outlined by the black square in (**a**). Bars = 1 μm in (**a**) and 0.5 μm in (**a’**). (**b**) 3D ET images of the intranuclear inclusion body in Y and X rotations obtained by ET. (**c**,**d**) 3D ET images of the virus-like particles in the Y and X rotations of different cross-sections obtained by ET. (**e**,**f**) 3D ET images of the virus-like particles (arrowheads) in the Z-slice position of different cross-sections obtained by ET. The bars in (**b**–**f**) are 100 nm, and those in (**c**,**d**) are 50 nm.

We next performed TEM of the images of a cluster of virus-like particles within the nucleus of the infected cell, which appeared to coincide with traces of intranuclear inclusion body filaments, as illustrated in Figure 3a. Figure 3a’ reveals a higher magnification micrograph of the region outlined by a black square in Figure 3a. The original video file of the 3D (Y- and X-directions) images obtained by ET is available as Supplementary Video S3. The 3D images on the Y- and X-axes in Figure 3b represent the images of clusters of virus-like particles that appeared to correspond to traces of filaments within the nuclear inclusions. Figure 3c and Figure 3d show the images of the clusters of virus-like particles obtained from the best shot to identify certain structures, demonstrating the surface and internal structure of the virus particle observed as viral particles with a diameter of ~100 nm, respectively.

The original video file of the 3D (Z-slice position) images obtained by ET is available as Supplementary Video S4. A representative 3D image of the virions on the core surface is depicted in Figure 3e. The cross-sectional structure observed inside a virion in the 3D images obtained by ET is illustrated in Figure 3f. These findings indicate that the virus-like particles are arranged in a configuration consistent with that of an intranuclear inclusion body.

### 2.3. Analysis of Intranuclear Inclusion Body and Viral Particles by Immunoelectron Microscopy

**Figure 4:**
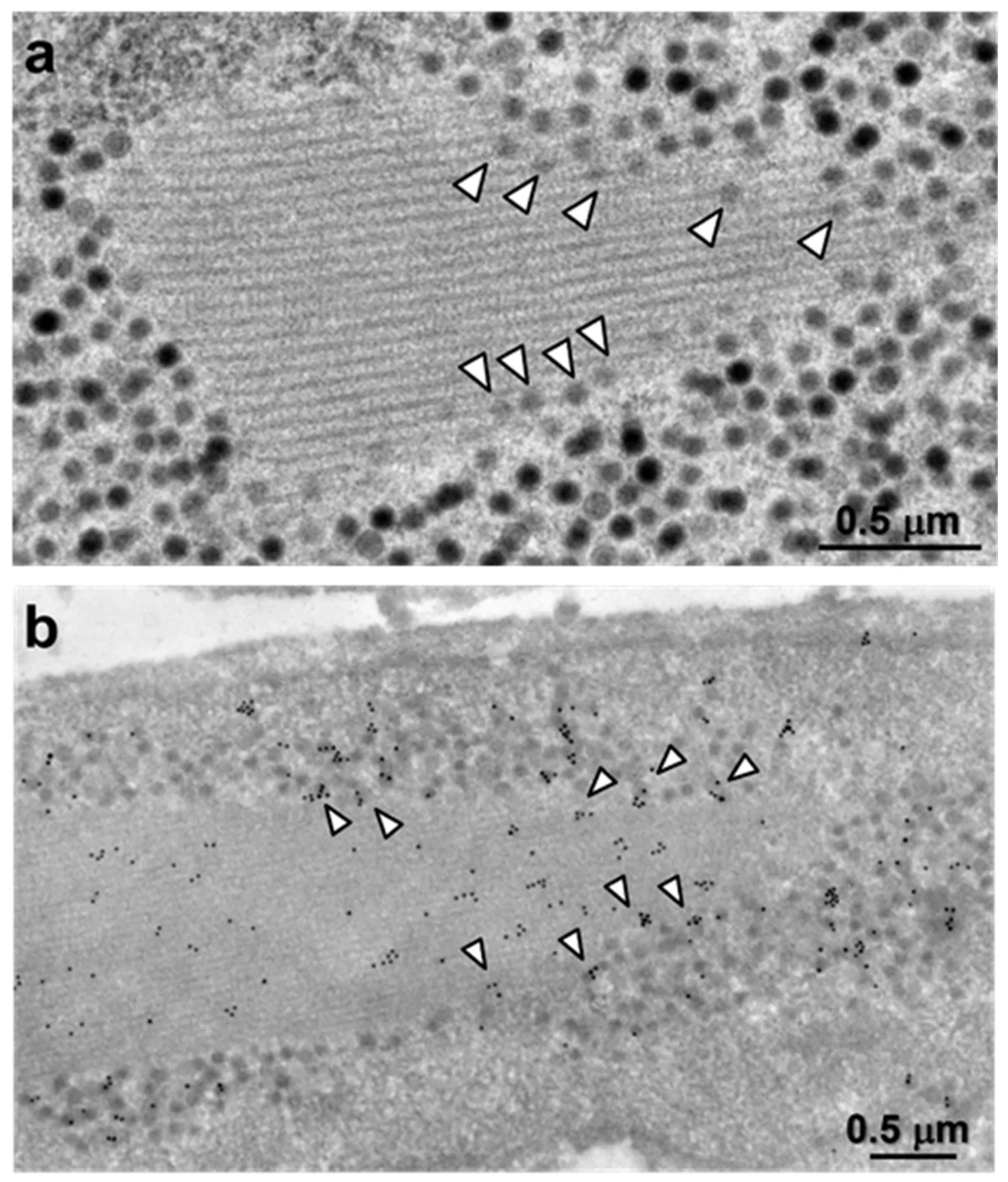
Intranuclear inclusion bodies in HAdV-5-infected Vero cells were compared using epoxy and Lowicryl K4M resin by TEM and immunoelectron microscopy. (**a**) Intranuclear inclusion bodies and surrounding viral particles (arrowheads) were observed by TEM in cells embedded in epoxy resin. (**b**) The localization of the hexon protein was observed in the intranuclear inclusion body containing the viral particles (arrowheads) in the interrupted region of the intranuclear inclusion body, and the virus-like particles were localized around the intranuclear inclusion body. Bars = 0.5 μm in all images.

To clarify whether the intranuclear inclusion body contains components of the adenovirus and whether the virus-like particles are indeed adenoviruses, we investigated the localization of the hexon protein, a constituent protein of HAdV-5, in the intranuclear inclusion body and HAdV-5-infected Vero cells by immunoelectron microscopy. To facilitate interpretation of the immunoelectron microscopy images, we first present a TEM image of the intranuclear inclusion body and viral particles in HAdV-5-infected Vero cells embedded in epoxy resin, which is unsuitable for immunolabeling (Figure 4a). Viral particles were detected within intranuclear inclusion bodies, as indicated by the arrowheads in the images. We then examined the localization of hexon protein by immunoelectron microscopy (Figure 4b). Hexon labeling was detected in intranuclear inclusion bodies, including regions containing viral particles, as well as in virus-like particles localized around the intranuclear inclusion body, as indicated by the arrowheads in the images. Viral particles within intranuclear inclusion bodies were detected by immunostaining with anti-hexon antibodies, as indicated by the arrowheads in the images. Hexon-positive viral particles were also detected in the nucleus, including in regions outside the areas surrounding the inclusions.

### 2.4. Analysis of the Association of Viral Particles with Intranuclear Inclusion Body

**Figure 5:**
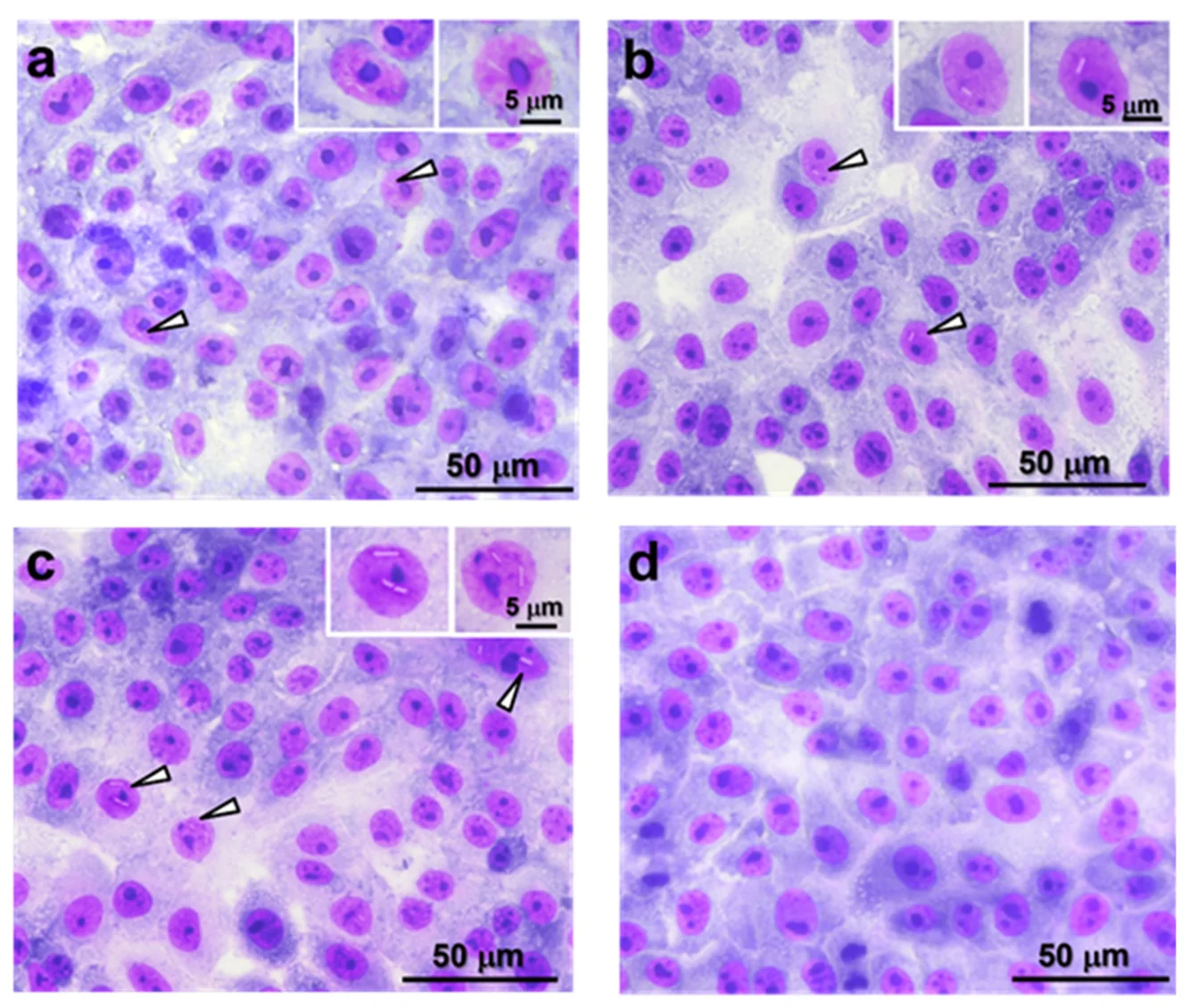
Time-course analysis of intranuclear inclusion body formation in HAdV-5-infected Vero cells by light microscopy after Giemsa staining. Light microscopic images of Giemsa-stained Vero cells (arrowheads) depicting intranuclear crystal structures at 14 h (**a**), 18 h (**b**), and 24 h (**c**) after HAdV-5 infection. Noninfected Vero cells are shown in panel (**d**). Insets depict enlarged views of the cells indicated by the arrowheads in panels (**a**), (**b**), and (**c**), respectively. Bars = 50 μm in all images.

To elucidate the relationship between the intranuclear inclusion body and viral particles, we first determined the stage at which the intranuclear inclusion body formed in HAdV-5-infected cells. No morphology of intranuclear crystal-like structures was detected in HAdV-5-infected cells up to 8 h, after which a small number of intranuclear crystal structures were detected in the 14 h (Figure 5a) and 18 h samples (Figure 5b), whereas numerous intranuclear crystal structures were detected in the 24 h samples (Figure 5c) using Giemsa counterstaining for optical microscopy. Figure 5d depicts the noninfected Vero cells.

**Figure 6:**
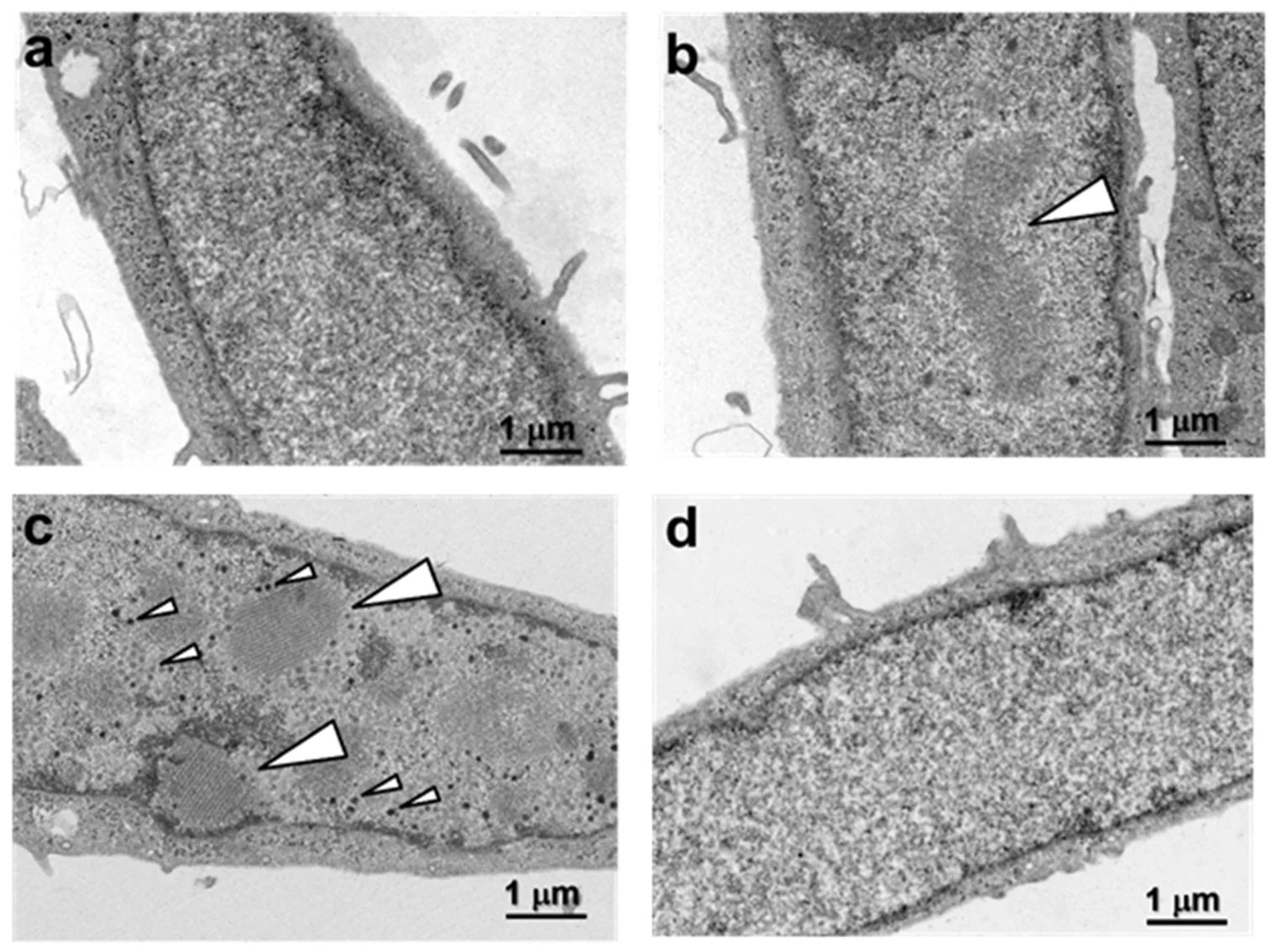
Time-course analysis of intranuclear inclusion body formation in HAdV-5-infected Vero cells embedded in epoxy resin by TEM. TEM micrographs of Vero cells showing intranuclear inclusion bodies at 8 h (**a**), 14 h (**b**), and 18 h (**c**) after HAdV-5 infection. Noninfected Vero cells are depicted in panel (**d**). The large arrowheads indicate intranuclear inclusion bodies and similar structures, and the small arrowheads indicate the virus-like particles surrounding the intranuclear inclusion body in (**c**). The insets depict enlarged views of the cells indicated by the arrowheads in panels (**b**) and (**c**), respectively. Bars = 1 μm in all images.

To confirm the intranuclear crystal-like structures observed at 14 h in HAdV-5-infected cells, the morphology of intranuclear crystal-like structures was observed by TEM in cells embedded in epoxy resin. Consistent with the findings of optical microscopy, no intranuclear inclusion body morphology was detected in HAdV-5-infected cells up to 8 h (Figure 6a), whereas a small number of intranuclear inclusion body-like structures (large arrowhead) were detected in the cells in the 14 h samples (Figure 6b). Moreover, in the 18 h samples, virus-like particles (small arrowheads) were detected around the intranuclear inclusion body (large arrowheads), as illustrated in Figure 6c, whereas no intranuclear inclusion body-like structures were observed by TEM in the noninfected Vero cell embedded in epoxy resin (Figure 6d).

**Figure 7:**
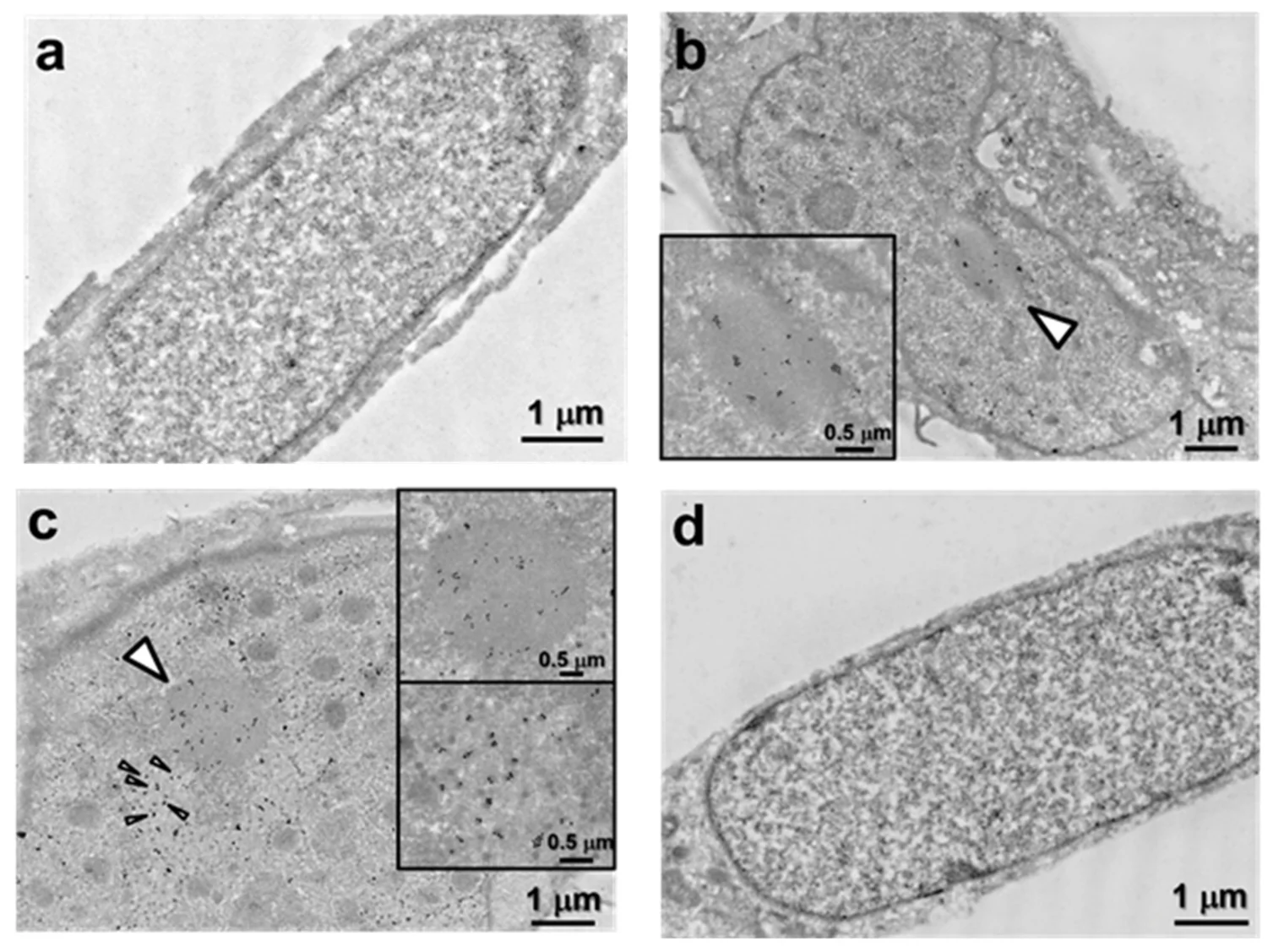
Time-course analysis of intranuclear inclusion body formation and viral particles in HAdV-5-infected Vero cells embedded in epoxy resin by immunoelectron microscopy. Immunoelectron microscopic micrographs of Vero cells at 8 h (**a**), 14 h (**b**), and 18 h (**c**) after HAdV-5 infection. Noninfected Vero cells are depicted in panel (**d**). The large arrowheads indicate intranuclear inclusion bodies and similar structures, and the small arrowheads indicate the virus-like particles surrounding intranuclear inclusion bodies in panels (**b**,**c**). The insets reveal enlarged views of the cells indicated by the arrowheads in panels (**b**) and (**c**), respectively. Bars in all images are 1 and 0.5 μm (inset of (**b**,**c**)).

To confirm whether the hexon protein was located in the intranuclear inclusion body-like structures and virus-like particles, we investigated the localization of the hexon protein in intranuclear inclusion body-like structures and HAdV-5-infected Vero cells by immunoelectron microscopy. No localization of the hexon protein was detected in the infected cells at 8 h (Figure 7a), whereas it was detected in the intranuclear inclusion body-like structures, indicated by the large arrowheads, in cells infected for 14 h (Figure 7b) and 18 h (Figure 7c) postinfection. Furthermore, the hexon protein was observed in the virus-like particles localized around the intranuclear inclusion body, as indicated by the small arrowheads in the images. Viral particles within intranuclear inclusion bodies were detected by immunostaining with anti-hexon antibodies, as indicated by the arrowheads in the images. Hexon-positive viral particles were also detected in the nucleus, including in regions outside the areas surrounding the inclusions. In contrast, no localization of hexon protein was detected in noninfected Vero cells by immunoelectron microscopy (Figure 7d). These data indicate the high possibility of intranuclear inclusion body formation before viral production.

## 3. Discussion

Morphological analysis of viral particles enables diagnosing several viral diseases. In particular, several unknown viral species have been compared with known viral species based on morphology. EM techniques, particularly TEM, are powerful tools for evaluating the morphology. Accordingly, in this study, we confirmed the presence of numerous viral particles near the nuclear inclusions, and viral particles in close contact with the inclusions were also detected by TEM. Through 3D ET, we identified viral particles within the intranuclear inclusion body and arranged in a crystalline structure parallel to the fibrous structures of the intranuclear inclusion body. Some particles were believed to be in the process of formation. We found that intranuclear inclusion bodies were present at a stage before viral appearance. Moreover, immunoelectron microscopy revealed no immune response to viral structural proteins in the early stages of infection; however, at 14 h of HAdV-5 infection, when the viral particles were not yet observed, anti-hexon antibodies of the viral protein reactions were detected against intranuclear inclusion body-like structures, and reactions were also observed against virus-like particles surrounding intranuclear inclusion bodies at 18 h. These findings suggest that the intranuclear inclusion body could have formed before the production of viral particles.

In our previous studies, using both TEM and 3D analytical techniques of ET, we examined and reported the 3D morphological structure of SARS-CoV-2 particles, reconstructed the images by ET [17,18,19], and obtained novel information on the ultrastructure and morphology of the virus. In the present study, using TEM and ET, we found that intranuclear inclusion bodies were closely associated with viral particles and revealed their 3D morphological relationship at a stage when the particles were believed to be undergoing formation, as reconstructed by ET. The reconstructed images demonstrated that virions protruded from the edges of the filaments within the intranuclear inclusion body and that these viral particles were embedded in the filamentous structure of the intranuclear inclusion body. Moreover, hexon, the most abundant structural protein of adenovirus, was used as a marker for viral components [20,21]. By applying an anti-hexon antibody, we examined the viral particles within infected cells and explored the presence of viral structural components in the intranuclear inclusion body. Immunoelectron microscopy revealed the presence of intranuclear inclusion bodies before the production of the virus. Our findings provide evidence that the formation and replication of viral particles may be closely associated with the use of intranuclear inclusion bodies.

**Figure 8:**
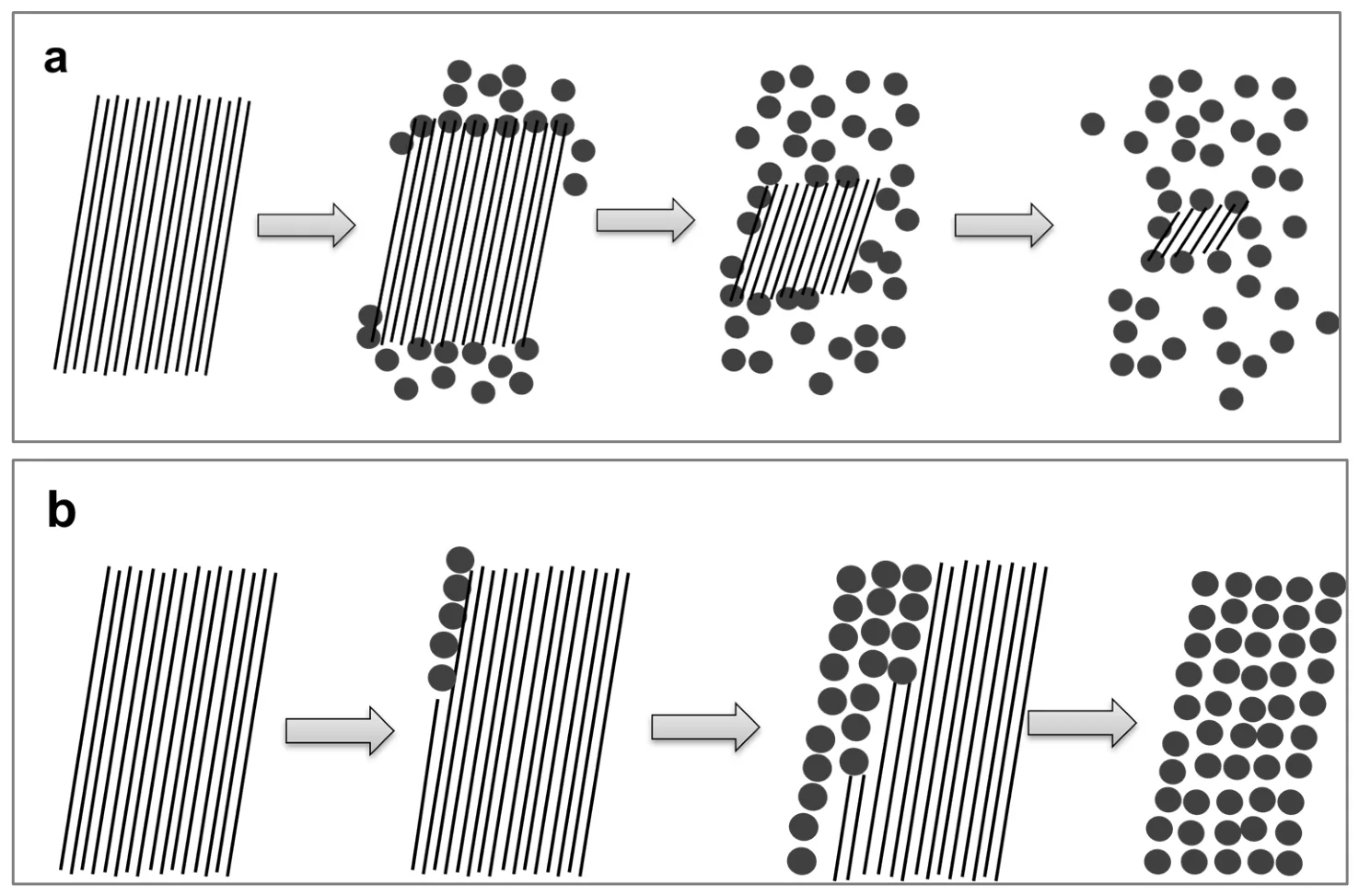
Illustrated figure of intranuclear inclusion body (lines) to viral particles (dots). Possibility 1: The intranuclear inclusion body serves as a scaffold for viral assembly and is gradually consumed during virion formation (**a**). Possibility 2: The intranuclear inclusion body serves as a template for viral assembly. Viral particles are generated around the inclusion body and become arranged in an outward direction (**b**).

Intranuclear inclusion bodies are observed during adenovirus infection and have been reported to participate in viral protein assembly [9,12,22,23,24]. Moreover, identifying viral particles within these nuclear inclusions is crucial for understanding the pathogenesis of adenovirus-associated diseases, including enteritis [25,26]. The production of viral particles is also closely associated with the formation of intranuclear inclusion bodies, and it has been proposed that viral replication and particle production occur within these structures [16]. Nevertheless, there have been no direct ultrastructural observations of this process using TEM. Therefore, we attempted to clarify the morphological process of viral particle production and its relationship with intranuclear inclusion body treatment. Our observations to date have consistently demonstrated numerous viral particles in close proximity to intranuclear inclusion bodies. Nonetheless, we did not observe the direct production of viral particles within the intranuclear inclusion body. Interestingly, viral particles were frequently arranged in crystalline arrays resembling the fibrous structures of the intranuclear inclusion body (Figure 3a); it may be possible that the viral particles overlap with or are embedded within these structures. On the basis of these findings, we propose two possibilities concerning the relationship between intranuclear inclusion bodies and HAdV-5. The first possibility, as illustrated in Figure 1b, is that intranuclear inclusion bodies themselves serve as a structural scaffold for the assembly of viral particles, with the viral particles aligning along their filamentous structures. As the viral assembly proceeds, intranuclear inclusion bodies are gradually consumed and eventually disappear from the viral assembly, as illustrated in Figure 8a. The second possibility, as depicted in Figure 3a, is that the intranuclear inclusion acts as a source of viral assembly, with the viral particles being generated around the inclusion and subsequently arranged in an outward direction, as illustrated in Figure 8b.

Regarding the intranuclear inclusion body, Pfitzner et al. [27] proposed that a membraneless intranuclear region termed the “late virion accumulation compartment” (LVAC) represents a type of nuclear inclusion body that facilitates the accumulation of viral particles. Similarly, Charman and Weitzman [28] characterized nuclear inclusion bodies as critical structures that serve as sites for viral DNA replication and packaging, thereby contributing to efficient viral propagation. The observations obtained from ultrathin sections examined by electron microscopy, together with the three-dimensional analyses performed using electron tomography in the present study, are consistent with these previous findings. We interpret these observations as morphological evidence supporting the existence and potential function of such nuclear compartments. However, because our immunological analysis in the present study was limited to the hexon protein, future studies, particularly the mechanistic studies planned in the near future, will require a broader panel of antibodies. By integrating detailed morphological analyses with molecular biological approaches, we aim to further elucidate the mechanisms underlying the formation and function of these nuclear compartments.

Although fluorescence microscopy is widely used to explore the functional aspects of virus–host interactions, electron microscopy, particularly TEM and ET, has proven invaluable for investigating the structural dynamics of HAdV-5-infected cells, as reported by Pfitzner et al. [29]. These approaches provide insights into the 3D ultrastructure of infected cells, which cannot be obtained by conventional fluorescence microscopy.

The findings of this study are based on analyses of HAdV-5. To achieve a broader understanding of adenovirus replication, it is essential to determine whether similar findings can be detected in other adenoviral types. Furthermore, extending this analysis to other viruses that form similar intranuclear inclusion bodies may uncover previously unrecognized mechanisms of viral replication and provide novel insights into the morphogenesis of DNA viruses.

Studies using TEM, particularly when combined with ET, play a vital role in clarifying the mechanisms underlying the formation of intranuclear inclusion bodies in HAdV-5-infected cells. These high-resolution imaging techniques enable the direct visualization of ultrastructural changes associated with viral replication within the host cell nucleus and their spatial relationship with intranuclear inclusion bodies, thereby providing valuable insights into the processes of viral replication and morphogenesis. In the present study, the TEM and ET analyses provide compelling morphological evidence that inclusion bodies are closely associated with adenoviral replication. Our observations suggest that these structures serve as reservoirs for viral components and are potential sites for the replication and assembly of the virus. Further ultrastructural analyses of viral propagation within infected cells may contribute to a more comprehensive understanding of the biological characteristics and replication mechanisms of ADV.

## 4. Materials and Methods

### 4.1. Cells and Virus

Vero (green monkey kidney cells, American Type Culture Collection #CCL-81, provided by the National Institute of Infectious Diseases, Tokyo, Japan) cells were cultured in Eagle’s minimum essential medium (EMEM; Nissui Pharmaceutical Co., Ltd., Tokyo, Japan) supplemented with 10% fetal bovine serum (FBS, Thermo Fisher Scientific, Waltham, MA, USA), 100 units/ml penicillin, and 100 μg/ml streptomycin (Nacalai Tesque, Inc., Kyoto, Japan) at 37 °C with 5% CO_2_.

HAdV-5 was propagated in Vero cell cultures. Infection titers of HAdV-5 were determined using a previously described chromogenic plaque assay [30]. Briefly, Vero cells were seeded in 24-well plates at a density of 1.5 × 10^5^ cells/well. The next day, the cells were incubated with serial dilutions of HAdV-5 samples in EMEM without FBS at 37 °C for 2 h. After removing the supernatant, the cells were cultured in EMEM containing 1% methylcellulose 4000 cp (FUJIFILM, Wako Pure Chemical Corporation, Osaka, Japan) and 2% FBS. At six days after infection, the cells were fixed with formaldehyde, permeabilized with Triton-X100 (Sigma-Aldrich, St. Louis, MO, USA), and incubated with a mouse anti-adenovirus monoclonal antibody (B025/AD51, Abcam, Cambridge, UK) at 4 °C. The next day, the cells were washed and incubated with alkaline phosphatase-conjugated anti-mouse secondary antibody (Cell Signaling, Danvers, MA, USA) at 37 °C. The virus plaques were visualized with BCIP/NBT substrate (Nacalai Tesque, Inc.), and the viral titer was calculated as plaque-forming units/mL.

Vero cells (1 × 10^6^) were seeded in a 25 cm^2^ cell culture flask and a one-well chamber glass slide 1 day before exposure to 1 × 10^6^ plaque-forming units of HAdV-5. After infection for 8, 14, 18, and 24 h, the HAdV-5-infected cells were trypsinized, pelleted for TEM and immunoelectron microscopy, and one-well chamber glass slides were fixed with methanol and then identified by Giemsa counterstaining for observation under a Nikon Ci-L optical microscope (Nikon Corporation, Tokyo, Japan).

### 4.2. Antibodies

In immunoelectron microscopy, a rabbit monoclonal antibody against the HAdV-5 hexon (ab316852, Abcam) was used as the primary antibody, and 10 nm immunogold-labeled anti-rabbit goat IgG antibodies (EY Laboratories, San Mateo, CA, USA) were used as secondary antibodies.

### 4.3. Fixation and Preparation of Ultrathin Sections and Staining for Microscopy and Immunoelectron Microscopy

Pelleted HAdV-5-infected cells were fixed with 2% glutaraldehyde in 0.05 M cacodylate buffer (pH 7.3) at 4 °C for 3 h, followed by washing five times with 0.05 M cacodylate buffer and postfixation with 1% osmium tetroxide in 0.06 M cacodylate buffer for 2 h. The samples were then dehydrated in ethanol solutions at serially graded concentrations, embedded in epoxy resin (Nisshin EM; Tokyo, Japan), and processed using routine EM procedures. Ultrathin sections (80 or 100 nm thickness) were prepared using a Reichert–Nissei ultramicrotome (ULTRACUT-N, Nissei Sangyo, Tokyo, Japan) and mounted on a nickel grid (300-mesh) supported by a carbon-coated collodion film. Ultrathin sections were subjected to conventional double staining with 4% (*w*/*v*) uranyl acetate for 10 min and Reynolds’ Pb for 3 min.

For immunoelectron microscopy, another sample was fixed with 1% glutaraldehyde in 0.05 M cacodylate buffer (pH 7.3) at 4 °C for 60 min, followed by washing five times with 0.05 M cacodylate buffer and dehydration in serially graded ethanol. The samples were then embedded in Lowicryl K4M resin (Polyscience, Warrington, PA, USA). Polymerization was allowed to occur in an ultraviolet irradiator (Dosaka EM, Kyoto, Japan) at −30 °C for 2 days and then at room temperature for 2 days. Ultrathin sections were cut using the Reichert–Nissei ultramicrotome and mounted on a nickel grid (300-mesh) supported by a carbon-coated collodion film.

The ultrathin sections on the grid were treated with 5% normal goat serum in 0.15 M PBS (pH 7.2) to block nonspecific reactions, followed by staining with a mouse antibody against the HAdV-5 hexon (primary antibodies) and 10 nm gold-labeled anti-rabbit IgG-labeled goat antibodies (secondary antibodies). The sections were first reacted by adding drops of the primary antibodies at room temperature for 120 min and washed five times with PBS. Then, they were allowed to react with the secondary antibodies at room temperature for 60 min and subsequently washed five times with PBS. The immunostained sections were fixed with 1% glutaraldehyde in 0.05 M cacodylate buffer for 15 min and washed five times with distilled water. Finally, the sections were contrast-enhanced with a mixture of ruthenium red and osmium tetroxide solution and stained with uranyl acetate aqueous solution, as described previously [31]. All procedures for contrast enhancement were performed at room temperature.

### 4.4. EM Observation and 3D Image Reconstruction

All sections were observed by TEM and ET (HT7800 type; Hitachi, Tokyo, Japan). The accelerating voltage for the EM observations was set to 100.0 kV. Electron micrographs of 80 nm thick ultrathin sections were obtained using a CCD camera (XR-81) (Advanced Microscopy Techniques Corp (AMT), Woburn, MA, USA) at a magnification of 3000–10,000×. The micrographs obtained by EM using the CCD camera measured 322 ppi and 3296 × 2472 pixels in size. Tilted images of a 100 nm thick ultrathin section were obtained using a TEM system equipped with a ±70° automated tilting apparatus, in which the angle of sequential images was tilted in steps of 2°. Sequential tilted images were reconstructed using the tomography-based reconstruction (TBR) method and 3D reconstruction software (Ver.07.21, EMIP-EX, Hitachi, Tokyo, Japan). TBR enables high-precision ET with few artifacts by suppressing the effect of missing information attributable to a limited sample tilt angle. The ET process has been described in detail by Wu et al. [17]. Electron beam tomography is conducted using a computer that processes a series of images formed by consecutively tilting the specimen through a specific tilt range. The PC-controlled 3D reconstruction system incorporates a function for the automatic or manual image acquisition of a tilted specimen and software for the 3D reconstruction of the acquired tilt series. The automatic or manual tilted specimen image acquisition function was activated to record the TEM images along with automatically correcting the position, focus, and magnification of the specimen. Furthermore, EMIP-EX contains a set of functions for aligning the tilt-axis position (X- and Y-axes and rotation) for each consecutively tilted or tilt-series image and calculating the 3D reconstruction data. Automatic or manual image alignment of a tilt-series image using EMIP-EX was performed in a non-marker-based manner. Alignment using EMIP-EX was performed along with coarse alignment using the image correlation method and fine alignment using the corresponding point search method. Fine alignment was conducted after extracting the corresponding points from each image, either automatically or manually. The distribution of the 3D shapes and different cross-sectional faces was displayed according to the calculation results (Hitachi High-Tech Corporation, Tokyo, Japan).

## 5. Conclusions

Intranuclear inclusion bodies were closely associated with viral particles. ET reconstruction revealed their 3D morphological relationship at a stage when viral particles were believed to be forming. The reconstructed images demonstrated that virions protruded from the edges of the filaments within the intranuclear inclusion body and that these viral particles were embedded in the filamentous structure of the intranuclear inclusion body. Moreover, immunoelectron microscopy revealed the presence of intranuclear inclusion bodies before viral production. Our findings provide evidence that the formation and replication of viral particles may be closely related to intranuclear inclusion body use. A more detailed analysis of the mechanisms underlying viral replication within infected cells may provide a deeper understanding of the biological characteristics of the virus.

## Data Availability

All data are included in this article and Supplementary Materials.

## Supplementary Materials

The following supporting information can be downloaded at: https://www.mdpi.com/article/10.3390/ijms27209181/s1.

## Abbreviations

The following abbreviations are used in this manuscript:

3D	Three-dimensional
HAdV-5	Human adenovirus type 5
TEM	Transmission electron microscopy
ET	Electron tomography
